# Population patterns in World’s administrative units

**DOI:** 10.1098/rsos.170281

**Published:** 2017-07-05

**Authors:** Oscar Fontanelli, Pedro Miramontes, Germinal Cocho, Wentian Li

**Affiliations:** 1 Universidad Nacional Autónoma de México, Facultad de Ciencias, México City 04510, México; 2 Universidad Nacional Autónoma de México, Instituto de Física, México City 04510, México; 3 The Feinstein Institute for Medical Research, The Robert S. Boas Center for Genomics and Human Genetics, Manhasset, NY 11030, USA

**Keywords:** population distribution, administrative units, power laws, Zipf’s law

## Abstract

Whereas there has been an extended discussion concerning city population distribution, little has been said about that of administrative divisions. In this work, we investigate the population distribution of second-level administrative units of 150 countries and territories and propose the discrete generalized beta distribution (DGBD) rank-size function to describe the data. After testing the balance between the goodness of fit and number of parameters of this function compared with a power law, which is the most common model for city population, the DGBD is a good statistical model for 96% of our datasets and preferred over a power law in almost every case. Moreover, the DGBD is preferred over a power law for fitting country population data, which can be seen as the zeroth-level administrative unit. We present a computational toy model to simulate the formation of administrative divisions in one dimension and give numerical evidence that the DGBD arises from a particular case of this model. This model, along with the fitting of the DGBD, proves adequate in reproducing and describing local unit evolution and its effect on the population distribution.

## 1. Introduction

The inhabitable area of the world is divided into politically distinct units, which may be countries or dependent territories. For the purpose of internal government and management, these units are often subdivided into smaller areas called administrative divisions or units. For example, the USA is divided into states (first-level division), which in turn are divided into counties (second-level division). China is divided into provinces and direct-controlled municipalities (first level), which are split into prefectures (second level), and prefectures are divided into counties (third level). Among other things, local administrative units can be seen as socially constructed entities, which serve as spatial scenarios for social and economic processes [1].

The heterogeneity of administrative units between and inside countries and territories is wide. Some countries, such as Grenade and Kiribati, have only first-level divisions. Most of them have divisions up to the second or third level, whereas some others have fourth-level or even smaller subdivisions. Furthermore, the structure and size of administrative units is a hallmark of a country’s internal organization. For instance, China reorganized its administrative unit system in view of the economic reforms in the decade of 1970, yielding economic benefits for the central cities [2]. By contrast, many developing countries have attempted to establish more decentralized local governments by increasing the number of administrative divisions [3]. These examples also illustrate the fact that territorial organization within countries undergoes constant evolution. Even in the absence of large administrative and political reforms, administrative units are constantly being created, destroyed, merged or split [4]. The high degree of diversity and complexity of the internal territorial administration of countries and territories worldwide relies partly on the population distribution over them [5].

A crucial factor for life within a given territory is its population, which does not distribute randomly over the available space [6]. For example, there are studies which show that a city’s population strongly correlates with many of the features of the city’s inhabitants: mean income, number of registered patents *per capita*, criminality rates, land value and rent prices [7,8]. The dependence of these quantities on city size has been mathematically quantified in what is called the *hypothesis of urban scaling*, which suggests that many properties of cities change with population in scale-invariant manners [9–11]. In this context, the study and understanding of the geographical distribution of the population within a given country or region becomes relevant, as it is a necessary step for the development of theories that could accurately describe the evolution of human agglomerations [12,13]. Regarding this matter, a natural question to ask is whether there is an equivalent hypothesis of scaling for administrative divisions.

Almost all studies regarding population distribution focus on city populations (see, for instance, [14–20]). There has been extended debate regarding whether city populations follow a power law or a lognormal distribution [21–23] and whether Gibrat’s law holds for city growth [24–26]). These two possibilities are classically sustained by Gibrat’s law (leading to a power law) [24,25] and by a Yule process growth (see, for instance, [27]), respectively. These studies often address the issue of finding an appropriate definition of the concept of a *city* or a *metropolitan area* [28]. However, the literature regarding the administrative unit population distribution is scarce. City boundaries can be vague, whereas administrative units are unambiguous. An administrative unit can encompass more than one city in some cases or just a fraction of a city in other cases. Administrative units are inclusive, whereas cities cover only a subset of the population. By definition, cities are populated places, whereas administrative divisions cover the whole country or territory and often include regions with very low population or regions that are not populated at all. Some examples of studies regarding population distribution in administrative divisions were given in [29], in which the authors analyse the population distribution for administrative divisions in China, Mexico and Spain.

In the present work, we perform a much more comprehensive study. Does a power law hold for the administrative unit population as it does for big cities? Are there any traces of ubiquitous behaviour? What is the effect on the population distribution of external agents delineating artificial boundaries for administrative territories? In this paper, we seek to answer these questions by addressing two main issues: (i) we provide a description and characterization of population distribution for secondary administrative divisions (SAUs from now on) for a set of 150 countries; in particular, we discuss the validity of power laws and propose applying a two-parameter rank-size function to fit the data; and (ii) we propose a computational one-dimensional toy model to describe the process of administrative unit formation and development, from which our two-parameter representation arises. This is a very simplistic model in which many details are omitted, but it captures the two main mechanisms of administrative unit evolution: splitting of divisions with large population and merging of geographically adjacent units.

The rank-size function that we propose to apply is the two-parameter discrete generalized beta distribution (DGBD), which has been used to fit rank-size and rank-frequency observations of natural, social and even artistic phenomena [29–37]. Regarding population distributions, it has been used to fit the size distribution of natural cities [38]. There is extensive work with this function and its applications in social and economic phenomena in a series of papers [39–41]. For example, the authors prove that the economical size distribution of Italian cities and municipalities over a period of time is statistically well described by a particular case of the DGBD [39]; they use the DGBD to describe the population distribution and aggregated income tax distribution of Italian cities and regions and propose that a Yule process adapted to finite-size data is approximated by the DGBD [40]; moreover, they construct a generalization of the DGBD and propose a preferential-attachment-like process that leads to this rank-size function [41].

In addition to these proposed models to explain the apparent ubiquity of the DGBD, we mention multinomial processes [42], restricted subtraction of random variables [43] and birth–death processes [44]. To model the formation of SAUs, we implement a version of the *split–merge process*, a computational mechanism in which administrative units are created and destroyed by joining and dividing them, thereby emulating the role of governments delineating internal boundaries. This process was originally proposed in [45].

The structure of the paper is as follows: first, we review the DGBD function and derive some of its relationships with some common probability distributions. Next, we analyse the population distribution in the population of SAUs for 150 countries, evaluate the goodness of fit of the DGBD and compare its performance with power laws. Then, we take the countries and dependent territories for which the DGBD is preferred over a power law and use the fitted DGBD parameters to construct a characterization of them according to the manner in which people internally spread. After that, we use the split–merge process to simulate the basic mechanisms of SAU formation and give numerical evidence that it leads to population distributions consistent with the DGBD function. Finally, we discuss how the differences between cities and administrative units naturally motivate the split–merge process, its suitability for outlining the administrator’s role in defining arbitrary boundaries and the appropriateness of the DGBD in representing these phenomena.

## 2. The rank-size representation and the discrete generalized beta distribution function

It is said that a random phenomenon presents a heavy tail when there is a relatively high probability for large, rare events to occur. When this is the case, it is customary to describe it via the rank-size or rank-frequency distribution instead of the probability density function (pdf) [46]. This is often the case for population distributions in which a small amount of highly populated regions encompass the majority of the population. Thus, we will adopt the rank-size representation to describe the population distribution within a country. First, we introduce this representation.

Let *X* be a continuous random variable with density *f*(*x*) and *X*=(*x*_1_,…,*x*_*N*_) be a sample of *N* independent realizations. The *i*-th order statistic *o*_*i*_(*X*) is the value of the *i*-th smallest observation; in this manner, *o*_1_(*X*) and *o*_*N*_(*X*) denote the minimum and maximum values of the sample, respectively. By writing *o*_*N*+1−*i*_(*X*)=*x*_[*i*]_, we obtain the ordered sample *x*_[1]_≥*x*_[2]_≥⋯≥*x*_[*N*]_. Intuitively, the rank of an observation is its location in this ordered sample (rank 1 corresponds to the largest observation, rank 2 to the second largest, etc.). Formally, the *rank* of the observation *x*_[*i*]_, denoted by *r*(*x*_[*i*]_), is the number of *x*′*s*≥*x*_[*i*]_. The statistic *R*=(*r*(*x*_[1]_),…,*r*(*x*_[*N*]_)) is called the *rank of the sample* *X*, and a plot of *R* against *X* is called a *rank-size plot* or *rank-size representation*. By construction, a rank-size plot must be non-increasing [47].

One method to link the rank-size representation with the density function is by noting that according to this construction, the rank of an observation *x* is proportional to the probability of making a larger observation [46], i.e. $r(x)\propto \int_{x}^{\infty }f(t)\;dt$. This leads us to define the *ranking function* of a real variable *x*, with respect to the density function *f*, as

2.1 $$r(x)=r_{m}+(r_{M}-r_{m})\int_{x}^{\infty }f(t)\;dt,$$

where *r*_*m*_ and *r*_*M*_ are the minimum and maximum ranks, respectively (usually, *r*_*m*_=1, and *r*_*M*_ equals the number of observations). For a sample of *N* observations of the random variable *X* with density function *f* and a particular observation *x*, the integer part of *Nr*(*x*) is the expected number of values greater than or equal to *x*. Therefore, the size versus rank plot, *X* versus *R*, approximates the ranking function in a similar manner as that in which the histogram approximates the density function. Note that for a given value *x*_[*i*]_, its rank *i* may have fluctuations in real data (in some samples, it can be the largest one, whereas in some other samples, it can be the second-largest one, and so on). However, the definition of the ranking function has the advantage of determining the expected value of the *i*-th observation *x*_[*i*]_ if the pdf *f*(*x*) is known. Note that according to equation (2.1), there is a relationship between the ranking of a number *x* and the cumulative distribution function evaluated at this same number, *F*(*x*)=*P*[*X*<*x*] because *r*(*x*)∝1−*F*(*x*). This also leads to the relationship *f*(*x*)∝*dr*/*dx*, implying that we can approximate the pdf of a random variable if we manage to approximate its ranking function, which we do with the rank-size representation (compute *r*=*r*(*x*), differentiate and normalize).

We use the term *rank-size function* to refer to a function that quantifies the dependence of an observation *x* on the rank *r*, that is, the inverse of equation (2.1), *x*=*x*(*r*). The most common example of a rank-size function is the power law, which is defined by *x*(*r*)=*A*/*r*^*α*^, where *A* is a normalization constant and *α* is a parameter. Note that the related pdf is also an inverse power function, *f*(*x*)∝1/*x*^1+(1/*α*)^. The DGBD rank-size function is a two-parameter function that outperforms other common distributions in describing scaling behaviours for a large variety of phenomena [29]. It is defined by

2.2 $$x(r)=\frac{C{\left(r_{M}+r_{m}-r\right)}^{b}}{r^{a}},$$

where *x* is the variable of interest, *b* and *a* are parameters to be estimated and *C* is a normalization constant. Unfortunately, it is not possible in this case to write its related pdf in a closed form, as it is not possible to analytically express the inverse function *r*=*r*(*x*), even though this inverse function exists. Still, there are methods to derive some relationships between the DGBD and common probability functions. First, we observe that the DGBD reduces to a power law when *b*=0. In particular, it is Zipf’s law when *b*=0 and *a*=1. Second, suppose that *X* is a random variable uniformly distributed over the interval (*α*,*β*). This means that *X* has the pdf *f*_*X*_(*x*)=(1/*β*−*α*)1_(*α*,*β*)_. Thus, for *x* in (*α*,*β*), equation (2.1) implies that

$$r=1+(r_{M}-1)\frac{\beta -x}{\beta -\alpha }.$$

Solving this last equation for *x*, we obtain, after some arrangement,

$$x=\frac{\beta -\alpha }{r_{M}-1}\left(\frac{(r_{M}-1)\beta }{\beta -\alpha }+1-r\right),$$

which reduces to the DGBD with *b*=1 and *a*=0 by taking *β*=*r*_*M*_/*r*_*M*_−1 and *α*=1/*r*_*M*_−1 in the limit when the maximum rank (and consequently the number of observations) *r*_*M*_ tends to infinity. Third, we observe that, for a zero-variance distribution located at *x*_0_ with a delta pdf function *f*_*X*_(*x*)=*δ*(*x*−*x*_0_), equation (2.1) reduces to *r*=*r*_*M*_, which is the DGBD with *b*=*a*=0 and a constant of normalization *C*=*r*_*M*_. Lastly, it has recently been shown that when *b*=*a*, the pdf associated with the DGBD can be analytically derived, yielding a novel probability law called the *Lavalette distribution*, which closely resembles the lognormal distribution [48]. In summary:

(i) a power law is represented by the DGBD with *b*=0. If additionally *a*=1, it is Zipf’s law;
(ii) a uniform distribution is represented by the DGBD with *b*=1 and *a*=0 in the limit when the number of observations is very large;
(iii) a point-located distribution is represented by the DGBD with *b*=*a*=0; and
(iv) when *a*=*b*, the DGBD represents a random phenomenon with a distribution approximately equal to a lognormal distribution.

Thus, the DGBD can be used to represent the population distribution in a completely ‘flat’ country, where all cities or administrative units have the same population (*b*=*a*=0) or, at the other extreme, in a completely disordered country in which population is distributed fully at random (a uniform distribution, for which *b*=1 and *a*=0).

## 3. Discrete generalized beta distribution and administrative unit population

**Figure 1.**
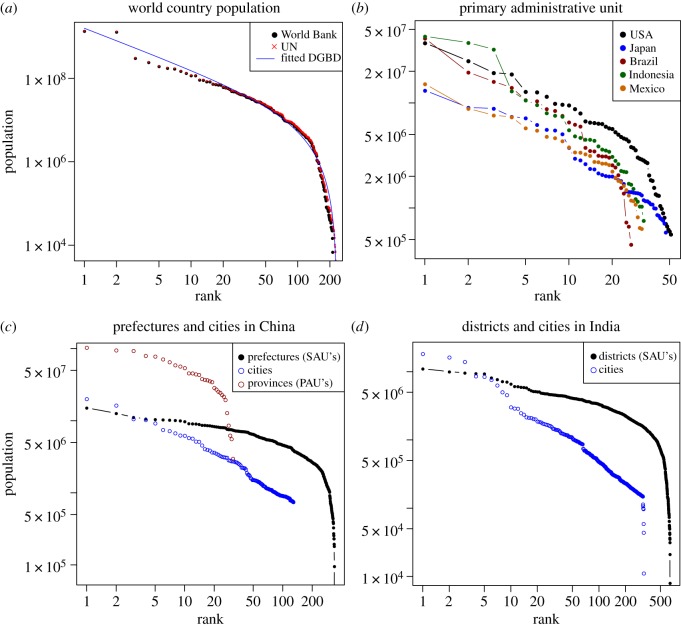
Examples of deviations from power laws. (*a*) Ranked population of countries and territories of the world according to data of the World Bank and the UN; the red line is the DGBD fit to the UN data. (*b*) Ranked population of primary administrative units in five different countries (states in the USA, Brazil and Mexico; prefectures in Japan; and provinces in Indonesia). (*c*) Ranked population of prefectures, cities and provinces in China. (*d*) Ranked population of districts and cities in India. All plots are on log–log scales.

Whereas city populations in different countries often exhibit power law or lognormal behaviours, when artificial divisions are considered, it is unclear what the distribution should be. To introduce the subject, we present in figure 1 several examples of ranked populations of administrative units. First, there is the distribution of the populations of all countries and territories of the world, which we can think of as zero-level administrative units (data from the United Nations (UN), https://esa.un.org/unpd/wpp/, and from the World Bank, http://data.worldbank.org/indicator/SP.POP.TOTL). As a matter of example of primary or first-level administrative units (PAUs from now on), we present the ranked populations of these units for five different countries. Because the number of PAUs is low for many countries, we will focus most of our analyses on secondary administrative units.

Figure 1*c* shows the ranked population for provinces and prefectures (primary and secondary administrative units, respectively) and for cities with more than 750 000 inhabitants in China. Even though cities and administrative units are different objects, in China, there is a certain correspondence between them: many administrative units are composed of a central city plus its neighbouring countryside, and whereas some cities form PAUs (the four largest ones), others constitute SAUs. These data are from the 2010 Population Census. Finally, we show ranked populations of districts (SAUs), agglomerations and cities in India; the data are from the 2011 Census of India.

Normally, power laws are expected when the rank-size or rank-frequency plot is approximately a straight line in the log–log representation. Although this is by no means sufficient statistical evidence to claim that a dataset or a phenomenon is a power law, it is a necessary condition that potential power law candidates must fulfil. Deviations from power law behaviour can be appreciated in figure 1. For instance, there is a breakdown in the tail of the country population distribution, yet the DGBD provides a good fit at both tails, as the blue line shows; these deviations are more difficult to appreciate in the PAU population but are more evident for SAUs, as figure 1*c*,*d* show. Note the deviation at *rank*=2 in figure 1*a*: here, we observe that the population of the second-largest country, India, is much above the fitted curve (we recall that the plot is on a logarithmic scale); such deviations are called *outliers* and are commonly observed in rank-size representations [39,49]. Regarding the distribution of city populations, we see in our examples that deviations from a power law appear in the low-population regime, but it is a good model for the largest cities.

We obtained SAU population data from the database Statoids (http://www.statoids.com, last consulted in April 2017; detailed information about the sources and dates of each dataset can be found on the aforementioned website), which gave us the SAU population for 150 countries and territories. We chose this as our global source because all datasets on this site come from official sources, which are not always accessible in a more direct manner. Whereas there are a few cases for which the data are more than 10 years old, for most of the countries, the information is from 2010 or later. Despite the fact that data within each country or territory were collected by its corresponding census or statistics office in different years, we still obtain a very general picture of the world population and its distribution at the current time.

To check the quality and reliability of these data, we compared our global source with three official data sources that were available to us: the 2011 census of Spain (http://www.ine.es/inebaseDYN/cp30321/cp_inicio.htm), the 2010 census of the USA (http://www.census.gov/topics/population.html) and the 2010 census of Mexico (http://www.inegi.org.mx/est/contenidos/proyectos/ccpv/cpv2010/Default.aspx). For the cases of Spain and the USA, the datasets from Statoids and the official sources are the same; in the case of Mexico, the data from Statoids are from the 2005 National Population Count (http://www.inegi.org.mx/est/contenidos/proyectos/ccpv/cpv2005/) and are not updated. The Statoids dataset includes 2455 total municipalities, whereas there are 2456 municipalities in the official database. The total population in the official database is 8.7% larger, accounting for the population growth between 2005 and 2010. The population of the largest municipality varies by 0.28% between the Statoids database and the official source. Note that the Statoids dataset for Mexico does coincide with the official data source, but it is not the most recent one. We performed our analyses on both datasets, the Statoids and the 2010 Census official source, and noted variations of 0.8% and 2.0% in the results (in the estimated *b* and *a* parameters of the fitted DGBD distribution); these examples indicate that the main results and conclusions of this work would not be substantially affected if we used other equally reliable data sources. An additional quality check we performed was to test the second-digit Benford’s law for each of our 150 datasets [50]. By performing a *χ*-square test on our data, Benford’s law failed to hold (with a *p*-value below 0.05) in only 11 cases (GNQ, GHA, IRN, MLI, MYT, RUS, SAU, TUR, UGA, GBR and WLF, in ISO3 country code).

From the 150 countries and territories we analysed, we took into account only those in which the number of SAUs is greater than 10, so we had a minimum number of points to perform a regression analysis. For each country, we ordered its SAU population by rank and fitted the data to the DGBD via a nonlinear regression of equation (2.2). To test the goodness of fit of the DGBD, we follow a procedure similar to that proposed in [51]. First, we computed the maximum vertical distance between the data and the best fit of the DGBD in the size versus rank representation. According to (2.1), rank is proportional to the cumulative distribution function; therefore, this statistic is proportional to the Kolmogorov–Smirnov (K–S) statistic, which is the maximum vertical distance between the empirical and theoretical cumulative distribution functions. This being said, we will call this maximum distance in the size versus rank representation the *K–S statistic* or *K–S distance*, but note that our statistic is only proportional to the actual Kolmogorov–Smirnov statistic. Next, we simulated a large number of DGBD data with the fitted parameters and computed the K–S statistic of each simulated sample. Our K–S statistic is a measure of the distance between the data and the reference distribution. We computed the fraction of simulated samples that are farther from the DGBD than our population data; this fraction gives an estimate of the *p*-value of the DGBD hypothesis. We recall that we cannot appeal to the distribution of the Kolmogorov–Smirnov statistic because our statistic is only proportional to the latter, which is why we used this resampling procedure. A larger *p*-value means random chances mostly lead to a worse fit, so our fitted function is not sufficiently bad to be rejected. On the other hand, a small *p*-value implies that our fitting performance is on the worse end among random chances and thus not good enough. With this method, the specific value of the estimation depends on some specific choices, such as how to manage samples with the same K–S distance, the number of replicates, etc. In particular, two countries with different number of SAUs may not be comparable in terms of their *p*-values because there is a tendency of the empirical *p*-value to be higher when the maximum rank of the data is low. Nevertheless, a large *p*-value still indicates that the fitted model cannot be rejected, after removal of countries with a very low number of SAUs.

The procedure to estimate the parameters *a* and *b* is the following: first, perform a traditional linear regression of the logarithmic transformation of equation (2.2). Then, use the fitted parameters to initialize a nonlinear regression of population *x* as a function of the rank *r* with the model given by equation (2.2) (see, for example, [52]). For the nonlinear regression, we used the Levenberg–Marquardt algorithm to minimize the sum of square of residuals; we chose this particular algorithm because it is more robust than Gauss–Newton [53]. The results of this nonlinear regression are our fitted parameters. The manner in which we chose the initial parameters for the nonlinear regression, via a linear regression, was to ensure quick convergence of the algorithm.

We took the countries for which the *p*-value is greater than 0.05 (insufficient evidence to reject the DGBD). We compare the DGBD model for these countries with a power law, which constitutes the traditional model for city population distributions. Note that the power law is a one-parameter model, whereas the DGBD has two parameters, so it is to be expected that the DGBD will produce better fits than the power law in the sense that the residuals will be lower. Consequently, the manner in which we will measure the quality of each model will be by balancing the fit to the data and the number of parameters. We use two approaches for this aim: first, we perform a likelihood ratio test (LRT; note that the power law is a special case of the DGBD). Here, a large *p*-value suggests rejecting the DGBD in favour of a power law. Following the suggestion in [54], we reject the DGBD when the *p*-value is greater than 0.001. A different approach is the use of the Akaike information criterion (AIC), which measures the relative quality of a statistical model and punishes models with more parameters. The model that exhibits a lower AIC=2k+Nlog⁡(RSS/N) is a better model (*k* is the number of estimated parameters, *N* the sample size and RSS the residual sum of squares). We recall that even though a power law is a particular case of the DGBD, they are different models from a statistical perspective, and the AIC takes into account the number of parameters of each model, thus measuring the relative quality of each one. There is only one case in which these two criteria do not coincide (Togo, where AIC favours the DGBD and LRT favours the power law; see table 1 in §6). After discarding all countries that have insufficient SAUs to perform a regression analysis, the sample reduces to 147 countries; 144 of them exhibit estimated *p*-values of the K–S test greater than 0.05 (Ethiopia, Mali and Niger are discarded). Out of the remaining 144 countries, the DGBD is preferred over a power law by both criteria in 141 cases (in Mayotte and Rwanda, both AIC and LRT favour a power law, whereas in Togo, there is no coincidence; see table 1 in §6). The results of these analyses are presented in full detail at the end of this paper in §7.

**Table 1.** Fitted *b* and *a* parameters, sample size (number of SAUs) *N*, *p*-value estimation for K–S test, AIC criterion between DGBD and power law (the AIC statistic is $\log({\text{AIC}}_{\mathrm{DGBD}})-\log({\text{AIC}}_{\mathrm{power}\;\mathrm{law}})$; a negative value indicates that the DGBD is preferred) and *p*-value for the likelihood ratio test (LRT) between DGBD and power law (a small *p*-value indicates that the DGBD is preferred).

country	*b*	*a*	*N*	K–S	AIC	LRT	country	*b*	*a*	*N*	K–S	AIC	LRT
Afghanistan	0.66	0.99	382	0.24	−455.37	0	Macau	1.76	0.44	7	0.44	0.84	0.005
Albania	0.58	0.88	61	0.42	−55.10	0	Madagascar	0.66	0.32	111	0.23	−155.42	0
Algeria	0.43	0.55	1541	0.72	−3068.41	0	Malaysia	0.35	0.61	144	0.38	−183.16	0
American Samoa	1.83	0.79	15	0.74	−7.69	0	Mali	1.40	0.23	50	0.01	−40.61	0
Argentina	0.60	0.71	511	0.43	−823.66	0	Malta	0.70	0.30	68	0.09	−91.97	0
Australia	0.74	0.54	654	0.15	−641.16	0	Marshall Islands	1.15	1.59	25	0.47	−4.23	3×10^−5^
Austria	0.83	1.55	95	0.16	−43.46	0	Martinique	0.83	0.78	34	0.51	−36.41	0
Bangladesh	0.41	0.45	64	0.40	−62.84	0	Mauritania	1.08	0.15	55	0.53	−67.41	0
Belgium	0.37	0.51	43	0.31	−24.13	0	Mayotte	0.04	0.95	16	0.54	4.51	0.677
Belize	0.75	0.27	12	0.46	−6.97	1×10^−6^	Mexico	0.58	0.56	2456	0.14	−3762.12	0
Benin	0.29	0.57	77	0.17	−19.45	0	Morocco	0.91	0.45	75	0.17	−95.22	0
Bhutan	1.03	1.16	262	0.18	−302.71	0	Mozambique	0.64	0.29	148	0.68	−258.41	0
Bolivia	0.63	0.91	112	0.91	−140.60	0	Myanmar	0.91	0.11	63	0.47	−94.98	0
Bosnia and Herzegovina	0.29	0.44	105	0.99	−150.06	0	Namibia	0.32	0.32	121	0.25	−166.95	0
Botswana	1.80	0.20	28	0.65	−33.61	0	Nepal	0.84	0.30	75	0.33	−83.06	0
Brazil	0.47	0.80	556	0.52	−629.25	0	Netherlands	0.37	0.64	504	0.81	−705.13	0
Bulgaria	0.47	0.98	262	0.41	−424.20	0	New Caledonia	0.44	1.14	33	0.59	−8.11	0
Burkina Faso	0.38	0.50	45	0.32	−29.62	0	New Zealand	0.66	0.59	74	0.83	−74.50	0
Burundi	0.24	0.23	129	0.69	−217.52	0	Nicaragua	0.58	1.15	153	0.25	−144.48	0
Cameroon	0.32	0.54	58	0.92	−44.25	0	Niger	0.78	0.19	37	0.03	−36.48	0
Canada	0.43	0.72	293	0.35	−329.85	0	Nigeria	0.26	0.29	775	0.61	−1694.98	0
Central African Republic	0.95	0.79	72	0.22	−67.95	0	Niue	0.73	0.59	14	0.33	−3.99	3×10^−5^
Chad	0.80	0.33	62	0.07	−56.57	0	Norway	0.48	0.90	431	0.61	−713.23	0
Chile	1.68	1.36	54	0.24	−63.42	0	Oman	0.79	0.52	61	0.37	−83.20	0
China	0.77	0.27	345	0.38	−810.67	0	Pakistan	0.60	0.38	30	0.15	−17.27	0
Colombia	0.70	1.13	1057	0.33	−1183.06	0	Palestine	0.80	0.21	16	0.88	−11.47	0
Congo	0.48	0.41	100	0.84	−135.53	0	Panama	0.49	1.10	76	0.70	−77.53	0
Costa Rica	0.44	0.49	81	0.67	−119.18	0	Papua New Guinea	0.15	0.35	87	0.31	−52.71	0
Côte d’Ivore	0.48	0.77	33	0.28	−12.09	0	Paraguay	0.37	0.74	224	0.92	−372.36	0
Croatia	0.46	1.40	556	0.27	−705.73	0	Peru	0.69	1.92	194	0.22	−201.04	0
Cuba	0.19	0.51	168	0.30	−134.79	0	Philippines	0.48	0.63	1634	0.69	−2559.73	0
Czech Republic	0.29	0.66	77	0.19	−14.45	0	Poland	0.24	0.57	379	0.30	−350.97	0
Denmark	1.04	0.56	99	0.09	−49.92	0	Portugal	0.35	0.59	308	0.23	−285.39	0
Dominican Republic	0.22	0.77	155	0.36	−157.19	0	Reunion	0.42	0.62	24	0.29	−5.78	5×10^−6^
Ecuador	0.59	1.02	216	0.66	−251.93	0	Romania	0.41	1.01	2951	0.30	−1273.87	0
Equatorial Guinea	0.50	0.88	30	0.80	−11.06	0	Russia	0.27	0.54	2581	0.21	−2172.22	0
Egypt	1.42	0.13	367	0.18	−687.37	0	Rwanda	0.02	0.15	30	0.27	1.96	0.023
El Salvador	0.42	0.61	262	0.30	−432.23	0	Sao Tome and Principe	0.32	0.87	7	0.65	3.42	0.098
Estonia	0.55	1.57	241	0.27	−95.11	0	Saudi Arabia	0.39	0.94	118	0.77	−85.43	0
Ethiopia	0.86	0.26	66	0.03	−78.56	0	Senegal	0.61	0.42	45	0.45	−51.00	0
Faroe Islands	1.01	1.49	34	0.46	−19.39	0	Sierra Leone	1.46	−0.08	15	0.23	−5.16	1×10^−7^
Fiji	1.00	0.64	15	0.74	−11.32	0	Slovakia	0.40	0.23	79	0.38	−117.57	0
Finland	0.41	1.19	69	0.37	−43.70	0	Slovenia	0.55	0.92	210	0.33	−263.93	0
France	0.41	0.35	96	0.20	−107.51	0	Solomon Islands	0.62	0.35	183	0.55	−283.63	0
French Guiana	1.39	0.59	22	0.88	−27.63	0	South Africa	0.63	0.39	52	0.85	−72.70	0
French Polynesia	0.66	0.54	49	0.13	−31.78	0	South Sudan	0.44	0.30	79	0.57	−93.72	0
Gabon	0.31	1.37	48	0.66	−20.67	0	Spain	0.60	0.67	52	0.68	−54.25	0
Gambia	0.33	0.93	37	0.95	−24.52	0	Sri Lanka	0.44	0.33	331	0.32	−581.19	0
Georgia	0.84	1.30	66	0.21	−44.94	0	Sudan	0.42	0.37	131	0.33	−150.25	0
Germany	0.34	0.53	402	0.29	−523.46	0	Suriname	0.97	0.31	62	0.07	−58.23	0
Ghana	0.20	0.66	110	0.23	−7.05	2×10^−6^	Swaziland	0.34	0.21	55	0.69	−69.74	0
Greece	1.24	0.63	326	0.20	−572.30	0	Sweden	0.33	0.75	289	0.70	−333.33	0
Greenland	1.11	0.68	19	0.40	−13.52	0	Switzerland	0.49	0.55	181	0.91	−291.06	0
Guadeloupe	0.72	0.46	32	0.91	−35.21	0	Syria	0.75	0.67	61	0.42	−25.96	0
Guatemala	0.53	0.76	331	0.32	−484.16	0	Taiwan	1.29	0.37	22	0.29	−17.80	0
Guinea Bissau	0.55	1.05	39	0.28	−14.28	0	Tajikistan	0.48	0.53	75	0.62	−58.83	0
Guyana	1.09	0.74	117	0.27	−163.06	0	Tanzania	0.48	0.27	129	0.70	−246.05	0
Haiti	0.43	1.20	42	0.27	−6.34	3×10^−6^	Thailand	0.36	0.33	926	0.09	−1362.43	0
Honduras	0.38	0.90	282	0.42	−326.14	0	Timor Leste	0.43	0.43	65	0.91	−85.28	0
Iceland	0.71	1.51	79	0.67	−97.43	0	Togo	0.37	1.06	35	0.28	−0.24	0.002
India	0.95	0.24	638	0.17	−1270.03	0	Tonga	0.41	0.67	23	0.31	−7.35	1×10^−6^
Indonesia	0.47	0.43	497	0.08	−623.93	0	Tunisia	0.41	0.23	263	0.08	−482.35	0
Iran	0.48	0.84	252	0.32	−218.17	0	Turkey	0.28	0.51	923	0.09	−902.32	0
Isle of Man	0.31	1.15	24	0.55	−1.70	4×10^−4^	Uganda	0.54	0.36	160	0.14	−200.82	0
Israel	1.10	0.25	15	0.23	−10.35	0	Ukraine	0.22	0.60	678	0.79	−501.90	0
Italy	0.33	0.55	110	0.99	−121.37	0	United Kingdom	0.40	0.33	406	0.34	−379.09	0
Japan	0.55	0.80	1180	0.34	−1443.85	0	United States	0.53	0.64	3143	0.25	−5487.94	0
Jordan	0.15	0.73	89	0.22	−39.15	0	Vanuatu	0.72	0.66	62	0.60	−76.95	0
Kazakhstan	0.17	0.47	200	0.18	−135.78	0	Venezuela	0.48	0.70	336	0.75	−601.21	0
Kenya	0.55	0.37	70	0.28	−89.20	0	Vietnam	0.65	0.18	661	0.90	−828.92	0
Lebanon	0.56	0.44	26	0.25	−18.63	0	Virgin Islands US	1.87	0.17	20	0.36	−23.04	0
Lesotho	0.56	0.74	129	0.18	−86.26	0	Wallis and Futuna	0.32	0.22	5	0.78	1.92	0.013
Liberia	1.03	2.54	136	0.14	−143.22	0	Yemen	0.67	0.29	333	0.24	−709.93	0
Lithuania	0.58	0.87	60	0.23	−23.37	0	Zambia	0.45	0.69	74	0.23	−47.68	0
Luxembourg	0.30	1.00	105	0.48	−77.50	0	Zimbawe	0.37	0.64	63	0.32	−33.52	0

**Figure 2.**
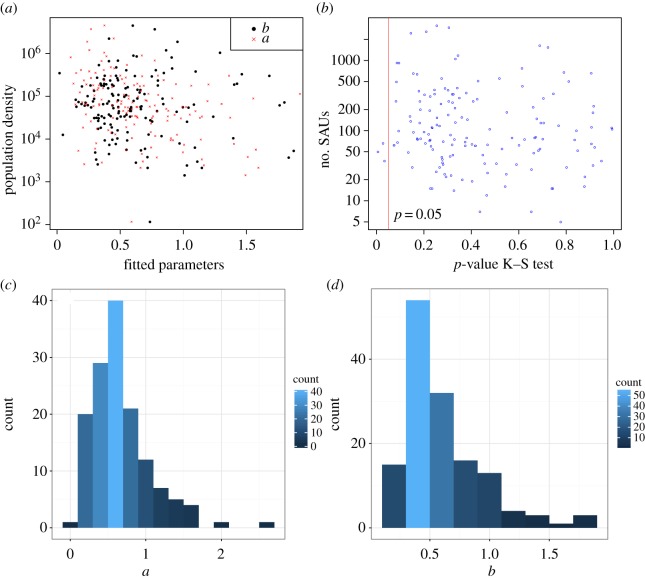
Fitted parameters, sample sizes, population density and goodness of fit. (*a*) shows on a semi-logarithmic scale the fitted parameters versus population density for the 150 countries and territories we analysed. (*b*) is a semi-logarithmic plot of the *p*-value for the K–S test versus the number of SAUs for the 150 countries; left of the red line is the rejection zone (*p*<0.05). (*c*) and (*d*) are histograms of the fitted *a* and *b* values for the 141 selected countries.

After these selection criteria, a set of 141 countries remains.The mean and standard deviation of the sample size (number of SAUs in these 141 countries) are 278.4 and 510.9, respectively. The mean and standard deviation for the fitted *b* parameter are 0.61 and 0.34, whereas the mean and standard deviation for the *a* parameter are 0.66 and 0.39, respectively. It can be seen from equation (2.2) that along the small-rank regime, the parameter *a* has a more marked effect on the shape of the distribution than *b*, which has a greater repercussion when the rank is high. This means that *a* describes the highly populated-unit regime (the tail of the distribution), whereas *b* characterizes the behaviour of the low-population units. Together, they give a sense of the internal arrangement of population across municipalities within a given country or territory. Another crucial variable for describing this internal arrangement is the population density, which involves the number of inhabitants and the available space, so a natural question to ask is whether there is a correlation between the DGBD parameters and population density. We show in figure 2*a* a plot of the fitted parameters against population density on a semi-logarithmic scale. A visual inspection of this plot and the values of the linear correlation coefficients (0.05 for *b* and population density, −0.22 for *a* and density) together suggest that there is no relationship between the fitted parameters and density. We wonder whether the goodness of fit of the DGBD is sensible for the sample size, i.e. the number of SAUs in each country or territory. We chose the *p*-value of the K–S test as a measure of the goodness of fit (there is no clear equivalent to the coefficient of determination for nonlinear regression; see [55] and references therein) and show in figure 2*b* a plot on a semi-logarithmic scale of this *p*-value against the number of SAUs. The linear correlation coefficient between these two variables is −0.11, which, jointly with visual inspection of the plot, suggests that there is no correlation. Figure 2*c*,*d* presents histograms of figure 2*a*,*b*, respectively, which indicate that these values are not randomly distributed but rather clustered around central values. There is one case, Sierra Leone, for which *a*<0 (*a*=−0.08). When this occurs, equation (2.2) fails to represent a rank-size distribution because it is not monotonic. However, the fits are still good; usually, this means that the maximum of the fitted curved is attained below *r*=1 [31]. To avoid this problem, we imposed the constraint *a*≥0 during the fitting procedure. Sierra Leone was the only case in which the estimation changed (*a*=0.00 after the constraint, whereas *b* remained equal).

We mentioned that outliers are commonly observed in rank-size representations. We recall that an outlier is an observation much above or below the extrapolation of the fitted curve [49]. Usually, this occurs over the low-rank regime, where the largest observations tend to be much greater than predicted by the model. This has been called the *king effect* when there is one outlier or the *king plus viceroy* effect when several outliers occur [39]. We wonder whether these kinds of deviations from the DGBD occur in our present analysis. They are not well appreciated in figure 10 because of the scale, but we show, for the matter of illustration, four examples in figure 9 in §7. Here, we show on a logarithmic scale the ranked population and respective DGBD fits for Egypt, Nigeria, India and the Philippines, all of them among the countries for which the DGBD is not rejected. We see that in Egypt, the king effect occurs, i.e. the largest observation is above the fitted curve, whereas in Nigeria, the king plus viceroy effect occurs. Interestingly, the outliers in India and the Philippines are in the low-rank regime, but they are not the largest observations. Additionally, some of them deviate below the fitted curve. Thus, in our case, outliers are not necessarily greater than predicted observations. We verified that these four datasets are still well described by the DGBD, according to our criteria, even after the outliers are removed.

**Figure 3.**
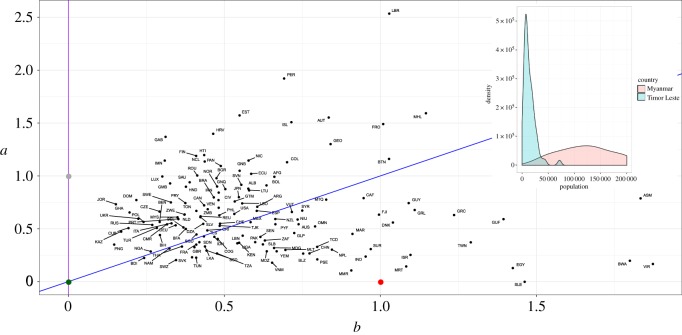
Scatter plot of fitted *b* and *a* for the 141 selected countries. Each country is indicated by its three-letter ISO3 code. The purple line corresponds to *b*=0, which represents a perfect power law; the blue line denotes *b*=*a*, which is the Lavalette rank-size function; the grey point is (0,1), a pure Zipf’s law; green is (0,0), representing a point-located probability distribution; and the red point is (1,0), corresponding to a random uniform distribution. We also show the density histograms for two countries: Myanmar, whose parameters indicate a closeness to a random uniform distribution, and Timor Leste, whose parameters suggest a distribution similar to a very narrow lognormal.

The fitted DGBD *b* and *a* parameters for these countries are shown in the scatter plot figure 3. Countries are indicated by their three-letter ISO3 codes. The grey, green and red dots represent idealized regions following perfect Zipf’s law, delta and random uniform distributions, respectively. We indicate with purple the vertical line *b*=0, representing perfect power laws, and with blue the line *b*=*a*, representing the Lavalette distribution, which, as we mentioned before, closely resembles a lognormal distribution. There has been debate about whether lognormal distributions are a better representation for city population than power laws. It might be said that, for SAU population, countries with *b*<*a* (between the blue and purple lines on the diagram) are somewhere between these two distributions, *b*≪*a* indicates that a power law fits the data well and countries below the blue line prefer a different model.

The scatter diagram allows a quantitative measure of the distance between the SAU population distribution within a country and an idealized Zipf’s law, a completely ordered or disordered population distribution, etc. We propose the Euclidean distance on the 〈*b*,*a*〉 plane to measure this. For example, Myanmar and Mauritania are close to being disordered (random uniform distribution), Burundi and Papua New Guinea are close to being delta-distributed, and countries such as Timor Leste and Nigeria are close to having a Lavalette distribution, which resembles a lognormal distribution. These observations are confirmed by observing the density histograms of Myanmar and Timor Leste, which are shown in figure 3. Indeed, we see that Myanmar has a very wide pdf, somewhat close to the constant pdf of a uniform random variable, whereas Timor Leste exhibits a taller and narrower pdf. The five countries and territories closest to being disordered or with an internal random uniform distribution are Myanmar, Mauritania, India, Israel and Palestine (note the geographical closeness of the pairs India–Myanmar and Israel–Palestine); the five countries and territories closest to being delta-distributed or flat are Burundi, Papua New Guinea, Nigeria, Swaziland and Namibia (note that four of them are in the African continent); the five countries closest to the Lavalette distribution are Timor Leste, Namibia, Nigeria, Burundi and Mexico. Finally, the five closest to exhibiting Zipf’s law are Luxembourg, Jordan, Dominican Republic, Gambia and Isle of Man.

**Figure 4.**
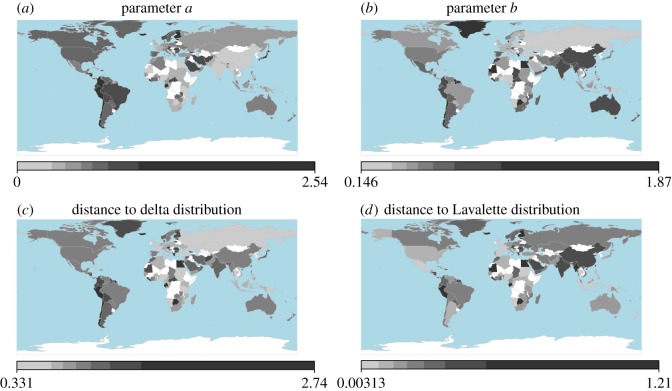
World maps of parameters and distances. (*a*) and (*b*) show the fitted *a* and *b* parameters, respectively, of the DGBD for the internal SAU population of each selected country and territory, (*c*) presents the Euclidean distance in the parameter plane 〈*b*,*a*〉 from a delta distribution in which all SAUs have the same population, and (*d*) shows the distance from a Lavalette distribution.

Even if the internal partition of a country is determined by a central administration, the SAU system might not be completely arbitrary, as there may be climatic and geographical factors constricting internal divisions and subdivisions. To investigate this issue, we present in figure 4 world maps showing the fitted *a* and *b* parameters of each country and Euclidean distances from a hypothetical country in which all SAUs have the same population (delta distribution) and a country with a Lavalette distribution. Note that the *b* parameter also gives the distance in this plane from a pure power law. Countries and regions shown in white are those for which the DGBD is not a good statistical model according to our tests or for which insufficient data were available. In these maps, we can see that there is indeed a certain correlation between geographical position and location on the 〈*b*,*a*〉 plane. For example, countries in East Asia are in general far from a power law compared with countries by the Guinea Gulf, which exhibit low *b* parameters. The *a* parameter dominates the low-rank/high-population regime and consequently the tail of the distribution; it is noticeable that almost every country and territory in the American continent has a relatively high *a* parameter, thus indicating a tendency of having very few highly populated places compared with most countries in Asia and Africa. In fact, we see some regional homogeneity within America, Europe and East Asia, whereas countries in Africa exhibit marked heterogeneity among them.

## 4. The split–merge model

Consider a delimited territory, a country, for example, in which the population is spread across communities, cities, towns, villages, etc. The population distribution of this aggregation may be well described by a Pareto distribution, a lognormal distribution or some other function, depending on the specific processes that drove the population growth and dispersion in the region. What occurs when a politician decides to create artificial boundaries, dividing the territory into well-separated administrative units? Certainly, we are now facing a different kind of object, and we do not know *a priori* if the distributions of populations in towns and artificial units are the same. It could happen that a big metropolitan area is disaggregated into several municipalities or that two different villages are grouped together into a common municipality. We simulated computationally one particular option for this mechanism by means of what we call the split–merge process:

(i) start with a sample ${\bar{X\;}\;}_{0}$ of *N*_0_ observations following some initial probability distribution *f*_0_. These observations represent populations of *N*_0_ human agglomerates; with these observations we create a one-dimensional array such that every agglomerate has two neighbours, except for the first and the last one, which are at the border and have only one neighbour;
(ii) pick the two largest values *X*^(1)^ and *X*^(2)^ of the sample and split each of them into two new values, *X*^(1)^→*p*_1_*X*^(1)^, (1−*p*_1_)*X*^(1)^ and *X*^(2)^→*p*_2_*X*^(2)^, (1−*p*_2_)*X*^(2)^, where *p*_1_ and *p*_2_ are random numbers on the interval (0,1);
(iii) randomly choose *q*% of the remaining observations and pair them with their neighbours; for example, pick *X*_*i*_ and replace it with the value *X*_*i*−1_+*X*_*i*_+*X*_*i*−1_, deleting *X*_*i*−1_ and *X*_*i*+1_ afterwards;
(iv) with the merged and split values and the remaining observations, construct the new sample ${\bar{X}}_{1}$ of size *N*_1_ following distribution *f*_1_; and
(v) repeat steps 2, 3 and 4 for *n* iterations.

**Figure 5.**
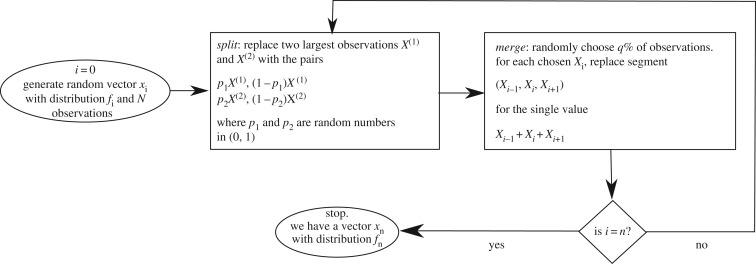
Flowchart of of the split–merge process.

This process is illustrated by the flowchart in figure 5. Step 2 simulates the process in which two large divisions are split into two administrative units each. Depending on the probability distribution from which *p*_1_ and *p*_2_ are sampled, we can simulate different methods to split a unit: for instance, if *p*_1_ and *p*_2_ follow a random uniform distribution, units are divided in an entirely arbitrary manner, resulting in the creation of many low-population units; on the other hand, if *p*_1_ and *p*_2_ follow a distribution with a peak at the centre (for example, a symmetric beta distribution), there is a higher probability for the divisions or municipalities to be split into more or less equal-sized units, thus simulating the division of a highly populated area into different units, each with substantially less population. This is typically what occurs when authorities want to improve the administrative efficiency in areas with rapid population growth. Step 3 simulates the action in which some neighbouring agglomerations are grouped into a single administrative unit or in which neighbouring units are merged into new, larger ones. By arranging the observations into a one-dimensional array, we impose a spatial constraint and ensure that only adjacent units are merged together or that new units split from a larger one end up being geographically close.

This model that we propose is a very generic one and does not consider historical and political factors, but its purpose is to give a framework for the simulation and quantification of the general processes behind administrative unit formation and evolution. Every country and region in the world has very specific circumstances that cannot be encompassed by any general simulation, yet this model has flexible elements, such as the initial distribution, the number of merged and split units in each step and the method of splitting the units, that can be changed to more realistically reproduce some features and behaviours of this very complex social and political phenomenon. We wonder what is the distribution of the municipalities populations after several iterations of this process. There are many options regarding how to proceed with this mechanism; for the present work, we chose two particular versions of the split–merge process to illustrate the idea.

**Figure 6.**
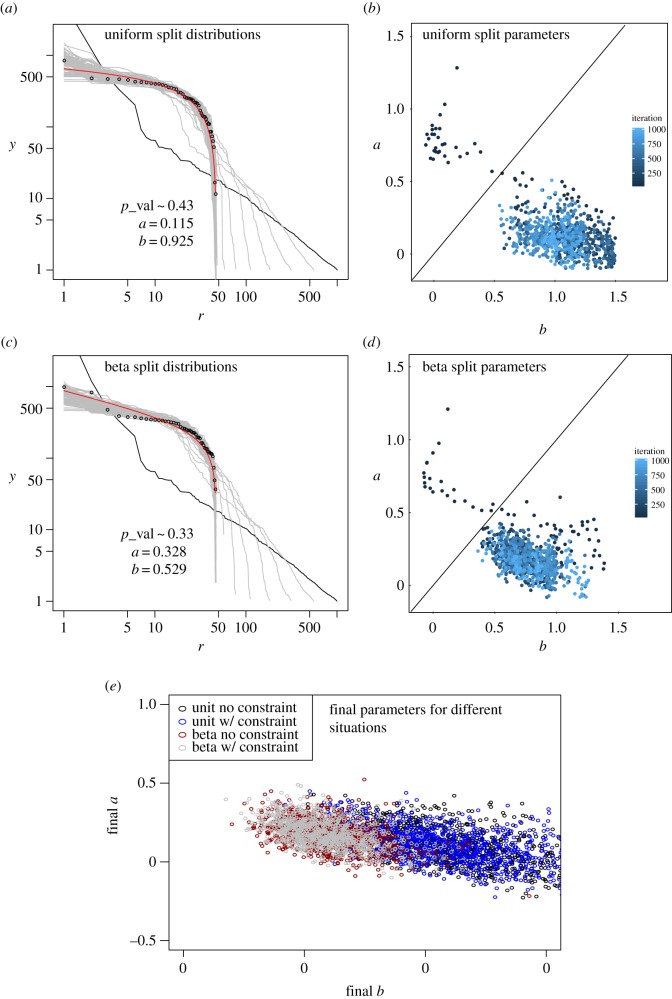
Realizations of the split–merge process in one-dimension. We see in black the initial rank-size function, in grey a sample of intermediate distributions, in circles the final distribution after 1000 iterations and in red the fitted DGBD to the final sample. The fitted parameters of the DGBD for each step of the process are also shown: (*a*) and (*b*) uniform split with spatial constraint; (*c*) and (*d*) non-uniform split with spatial constraint. We also display the *p*-values of the DGBD hypothesis for the final sample and fitted parameters. (*e*) The *b*,*a* parameters after 1000 iterations for 1000 different realization of four cases of the process.

We present results for two split–merge process realizations, both with the Pareto distribution as the initial distribution. We chose this initial distribution because it is a commonly accepted model of city population distribution. In each process, we started with *N*_0_=1000 initial agglomerations and iterated 1000 times. First, we chose uniform random numbers to split the large units, which has the effect of creating many small-sized municipalities; then, we sampled these numbers from a beta distribution *f*(*x*|*α*,*β*)∼*x*^*α*−1^(1−*x*)^*β*−1^ with *α*=*β*=2. This is a distribution with a maximum at *x*=0.5, so large units are split into equal- or similar-sized units much more often. In each iteration, we merged 3% of the remaining units. Figure 6 displays the results: in frames (*a*) and (*c*), we see the rank-size plot for the initial set, for a selection of 100 intermediate sets (one every 10 iterations) and for the final set after 1000 iterations. For the final set, we also show the fitted DGBD, in addition to the fitted *b*,*a* parameters and the estimated *p*-value of the DGBD hypothesis. To compute this *p*-value, we used the test described in the previous section. In frames (*b*) and (*d*), we show the temporal evolution of the parameters in each realization from early iterations (dark blue) to latter ones (light blue). We also wondered how significant is the spatial constraint for our results. To study this, we repeated the simulations with fixed random seeds but this time without the spatial constraint, not noting any significant change in the results with and without the spatial constraint [56]. Finally, we performed each of these four simulations 1000 times with different random seeds and registered the estimated *b*,*a* parameters for the final iteration; a scatter plot of them is provided in figure 6*e*.

The first thing to observe is that in neither case do we reject the DGBD, according to the estimated *p*-values. Apparently, the DGBD is indeed an adequate model for describing the distributions in this kind of process. Note how the parameters follow a well-defined trajectory on the 〈*b*,*a*〉 plane at first (dark blue), but begin to move somewhat erratically as times passes (light blue). We also note, how almost invariably, *b*>*a* for large times. As we mentioned in the previous section, the region *a*<*b* represents countries whose internal population distribution is somewhere between the power law and lognormal distributions. We speculate that these distributions break as the territory is artificially divided into municipalities, leading to new distributions for which *b*>*a*. Note, for instance, that figure 2*c* shows that the most common value for *a* is somewhere around 0.5 (the actual mode is between 0.50 and 0.52), whereas our model predicts lower values of *a* in the long run. According to these simulations, countries or territories move to the region *b*>*a* with time, yet we see in figure 3 many countries on the other side of the line. We speculate that these countries are in the process of breaking the initial lognormal-like or power-like distributions and will move towards *b*>*a* as their internal divisions are split and merged over time. This hypothesis is consistent with our simulations, in which there is a transition from one to the other side of the *a*=*b* line. The fact that sub-samples or aggregations of zipfian sets significantly deviate from Zipf’s law has already been observed [57]. Here, we have a new result in this direction: a perfect zipfian or lognormal set, describing population in natural agglomerations, transforms into a different kind of set of different kinds of objects, artificial municipalities with arbitrary borders, for which the initial distribution no longer holds. Rather than fixed values, the *a* and *b* parameters seem to have cluster-like attractors, which means that they end up moving in a bounded area when the number of iterations is very large. As figure 6*e* shows, these clusters are more extended when units are split with the uniform distribution and more localized when they are split with the beta, non-uniform distribution. Thus, the manner in which units are divided does have an effect on the final distribution, even though the corresponding clusters overlap. We note that the clusters for the processes with and without the spatial constraint clearly overlap, so the spatial feature of our model seems to have no effect on the final distribution.

**Figure 7.**
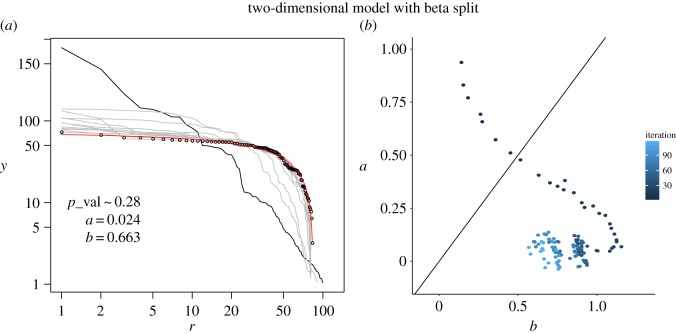
Realization of the split–merge model in two-dimensions. (*a*) Initial, intermediate and final distributions after 120 iterations, as well as the fitted DGBD for the final distribution. (*b*) The trajectory of the fitted DGBD parameters.

We implemented a two-dimensional version of the split–merge model by arranging the units in a hash table, giving each node a random population according to a certain initial distribution. The hash key is the ID of the unit or node, the population of the administrative unit is a hash value assigned to the ID and we use another value to specify an array of its neighbours. To do the split, we pick a node and create two new nodes, split the population in two, one for each node, according to a certain probability distribution and remove the original node. To do the merge part, we pick two neighbouring nodes, sum the population of each one and replace them with a single node with the total population. We show in figure 7 a particular realization of this process with an initial 10×10 unit grid with a Pareto distributed population, splitting the two largest units at each step with a symmetric beta distribution and merging 3% of the remaining units. Comparison between this figure and figure 6 suggests that results form the one-dimensional model and this particular two-dimensional implementation are not quite different: we still see the break-up of the initial power law into distributions in the *b*>*a* regime, with a cluster-like attractor, where *a* is very low. To study the behaviour of the two-dimensional model more carefully is the matter of future work.

This model mimics some of the more general mechanisms driving the evolution of administrative unit systems. As a matter of example, consider the history of regional divisions in Georgia [58], with a first stage of antiquity (the initial distribution in our model), followed by a unification of territories (the merge part of the model) and a feudal fragmentation (the split part). There are countries, such as China, where the division system is recent and results from central planned decisions [2]. On the other hand, there are other countries, such as Spain, where political divisions are very old and have suffered only minor changes [59]. Another interesting example is the case of France, where recently there were various plans to reduce the number of regions, finally reducing it from 22 to 13 after months of debate; this is an example of a directed reorganization with the aim of reducing bureaucracy and administrative costs, but with possible negative effects according to entropy or disorder criteria [60]. Every country has its individual historical, economic, social, political and geographical conditions determining the evolution of its administrative division system; as a consequence, any model attempting to simulate some features of this complex process must be both generic and flexible.

It is important to observe that the number of updates in the administrative system of any particular country or territory is limited by its own history; in terms of the split–merge model, this means that the number of iterations or steps may be quite short. As a consequence, we expect that the transient points in the dynamic of the model may be more relevant than the limiting point when time goes to infinity. Thus, we expect most countries and territories to be on the transient regime of the model, and not necessarily near the attractor with *a*≈0. This explains why the most probable value of *a* is around 0.5 in our data. This model simulates two of the main processes leading to the evolution of an administrative unit system, the merging and splitting of units, and is consistent with the previous statistical description of population distribution with the DGBD; we believe these results to be encouraging, and they open the path to extending this model to a more realistic simulation setting using a GIS-based model. A second aspect that deserves attention is the possibility of a derivation of an analytic formulation of this process; these two subjects are matters for future work.

## 5. Administrative divisions against natural cities

**Figure 8.**
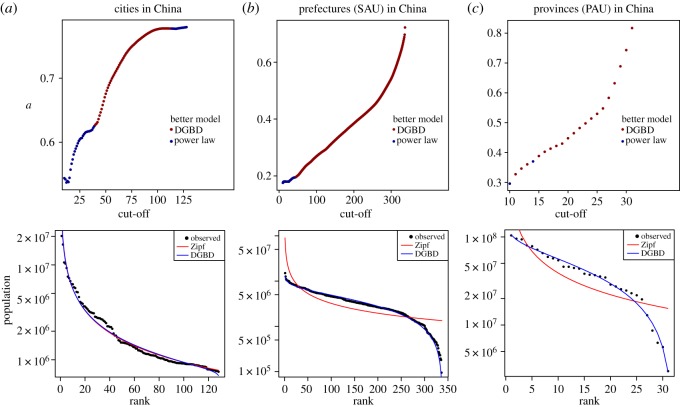
Effects of truncation in China’s population. Estimated exponent of power law versus sample size (taking the ‘cut-off’ largest units in the sample). According to the LRT, points marked in red and blue are better described by the DGBD and a power law, respectively. We also see the rank-size plot for the whole samples for the population distribution in China of its (*a*) cities, (*b*) prefectures and (*c*) provinces.

To further comprehend the differences between cities and administrative divisions, we compared population distribution for cities, primary (PAU) and secondary administrative divisions in a country from our sample. We chose China for this analysis because it is the most populated country in the world. The population data correspond to the 2010 Population Census by the National Bureau of Statistics of China. As we already mentioned, it has been observed that a power law fits city population data well for the upper tail (large populated cities) but fails when smaller cities come into consideration, so a cut-off is usually introduced [61,62]. We considered cities with more than 750 000 inhabitants (128 cities) and took sub-samples of the 10,11,…,128 largest ones, fitted each of them with a power law *x*(*r*)=*C*/*x*^*a*^ and estimated the exponent *a*. The purpose is to illustrate that statistical results are sensitive to cut-off procedures, so they ought to be performed with extreme care. Figure 8*a* shows the number of cities in the sample (the cut-off) versus the estimated exponent. We see that the parameter is very sensitive to the threshold value, so the decision of where to set it should be made with extreme care. This truncation may also introduce deviations from a power law; for each sub-sample, we tested the goodness of fit of the power law model against the DGBD via the LRT, rejecting the DGBD in favour of a power law when the *p*-value is less than 0.001, according to the suggestion of [54]. We show in red those sub-samples for which the DGBD is preferred over a power law at this level and in blue those for which a power law is better according to this criterion. We also show the rank-size plot for the whole sample of cities and its respective DGBD and power law fits. With these results, we speculate that truncation produces deviations from a power law that are well modelled by the DGBD. Aside from truncation, what occurs when territorial division comes into play?

The same analysis was performed for the prefectures’ (SAUs’) populations in China. We took several sub-samples by introducing cut-offs, with the goal of studying how sensitive the exponent of a fitted power law is to a cut-off value. In figure 8*b*, it is possible to see this sensibility. Except for a few number of small sub-samples, the DGBD is a better model than a power law on almost every occasion. Now, we are not only dealing with truncation but also with a process of city splitting, as there are natural cities divided into several administration units (for instance, the city of Shanghai is subdivided into 16 SAUs). The splitting mechanism also introduces deviations from a power law. Finally, we considered province (PAU) populations; see figure 8*c*. Note that the four largest cities in China (Beijing, Chongqing, Shanghai and Tianjin) are officially PAUs; for this analysis, we considered Chinese provinces without these four, so we can clearly distinguish them from natural cities. This time, the DGBD performs better for almost all sub-samples. There is a certain correspondence between cities and administrative units in China: some administrative units are formed by a large central city plus its neighbouring countryside, as is the case of the four largest cities; however, this correspondence ultimately breaks. For instance, the cities of Beijing, Chongqing, Shanghai and Tianjin, which have the status of direct-controlled municipalities, equivalent to that of provinces, are divided into 16, 38, 16 and 16 SAUs (districts and counties), respectively. This accounts for the splitting phenomenon; the effect of merging also becomes visible because there are some sets of cities grouped into the same unit (for example, the province of Anhui has 12 large cities). This merging operation also causes discrepancies between the observed distribution and power law. As we can see from the rank-size plot, the DGBD is a better model for the PAU population.

In summary, the DGBD is a better model than a power law for describing administrative unit population. Although there is an initial correspondence between cities and administrative divisions, this correspondence ceases to hold when large cities are divided into several units (split) and when separate units are grouped into larger ones (merge), yielding divisions with more than one large city or town. Cities and administrative units are clearly different objects, whose populations are described by different kinds of datasets and, in most cases, follow different distributions. Finally, these results suggest a method to formulate a hypothesis of scaling for administrative units.

## 6. Discussion and conclusion

In this work, we investigated the topic of the formation, evolution and population distribution of administrative units for different countries and territories around the world. Because administrative divisions are artificially defined, serving diverse political and administrative purposes, and are constantly evolving, a high degree of heterogeneity and diversity was to be expected. We tested the relative quality of the commonly used power law against the two-parameter DGBD rank-size function for describing the population distribution of second-level administrative units. We used a resampling approach to the K–S goodness of fit test to evaluate the fitting of a power law and the DGBD to the data and the AIC together with a LRT to compare the performances of these two models. Even though the DGBD has one more parameter than the power law, our tests measured the balance between the data fit and number of parameters of each model, yielding a fairer measure of their quality. According to our criteria, we concluded that the DGBD is a good model in 96% of cases; there are only two cases that exhibit a good power law (Mayotte and Rwanda, see table 1), representing 2% of the total, and in 2% of cases, neither is a good model. However, within those countries in which both models are rejected by the K–S test, the DGBD is a higher-quality model than a power law in all cases, considering the balance between fit and number of parameters. These results, together with visual inspection of figure 10, support the idea that the DGBD is an adequate choice when fitting administrative unit population data. As an additional analysis, we tested the performance of the DGBD and a power law as statistical models to fit world population by country, which can be thought of as a zero-level administrative unit. This is a case in which the DGBD is not rejected by the K–S test and is preferred over a power law in terms of both the LRT and AIC criteria.

The DGBD allowed us to propose a metric to characterize and compare the internal population distribution between different countries and territories. When fitting the data with the DGBD, if *b*<*a*, it is likely that the distribution is somewhere between the Pareto and lognormal distributions, as it is for cities and metropolitan areas. On the other hand, cases in which *b*>*a* call for a different distribution. We derived some analytic relationships between the DGBD and a uniform distribution, a delta distribution and a power law, and suggested a link to the lognormal distribution. This indicates that the DGBD is a very flexible function capable of providing a good representation of a large number of datasets. Consequently, it is a good candidate for characterizing and comparing population distributions in different parts of the world, as they could be following different dynamics, thus making it difficult to propose a more universal model.

Cities, urban areas and towns are often considered to be organic, natural agglomerations of people living together, whereas administrative divisions arise from a combination of these self-organized clusters from one side and local and central governments establishing boundaries from the other. The formation of administrative units is a process driven by controlling agents in addition to a certain degree of self-organization, exhibiting a high degree of complexity. The policies and procedures that different countries use to divide their territory have disparate purposes but are not completely arbitrary: in general, there is at least one city or town in each administrative division (the capital), large cities are often split into several distinct units, etc.

We proposed the split–merge process to simulate the action of bureaucrats and politicians partitioning the territory of a country into administrative units. Because some cities are divided into distinct units and some units have more than one city, the correspondence between cities and administrative divisions breaks, so we do not have the same population distribution for these two types of data. Computational simulations show how the initial power laws and lognormal distributions of cities evolve into other kinds of probabilistic laws for municipalities as the territory is partitioned; the distributions in latter stages are well represented by the DGBD with *b*>*a*. With our numerical evidence, we conjecture that DGBD rises form the split–merge process. Further analyses focusing on the details of the process and a possible analytic derivation of the DGBD are still needed.

In addition to the prior discussion, with these results, we extend the range of applicability of the DGBD function, contributing to the literature of its claim of ‘universality’. Furthermore, we use for the first time K–S tests and bootstrap methods in this context, which provide a measure of its goodness of fit. Our results also raise the question of the performance of the DGBD in describing populations in more ‘natural’ urban agglomerations.

In conclusion, the DGBD is a better statistical model than a power law for fitting administrative unit population data in most of our samples. Deviations from power laws and lognormal distributions arise as a consequence of a dividing and grouping mechanism. The split–merge process can satisfactorily mimic these mechanisms. The local administrative unit approach, the DGBD function and the split–merge model extend the research on population distribution and the undeniable role of artificial boundary settings.

## 7. Results of statistical analyses

**Figure 9.**
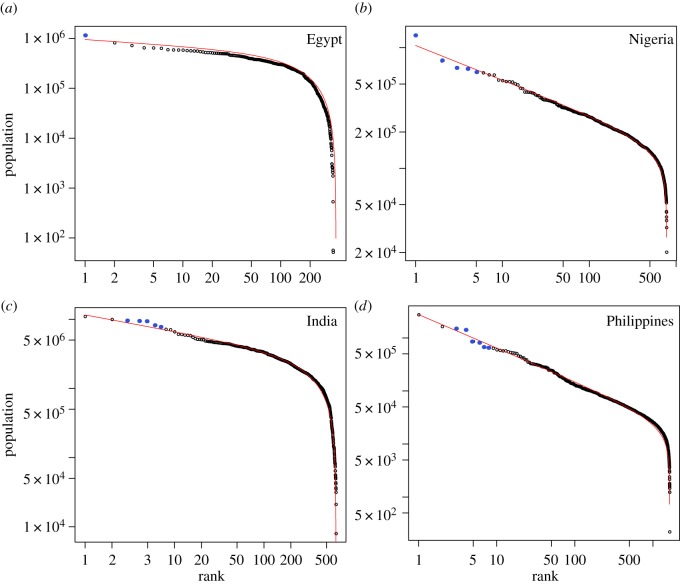
(*a*–*d*) Outliers, king effect and viceroy effect. Ranked SAU population and respective DGBD fits in log–log scale for four countries where the DGBD is a good model. Outliers are shown in blue.

**Figure 10.**
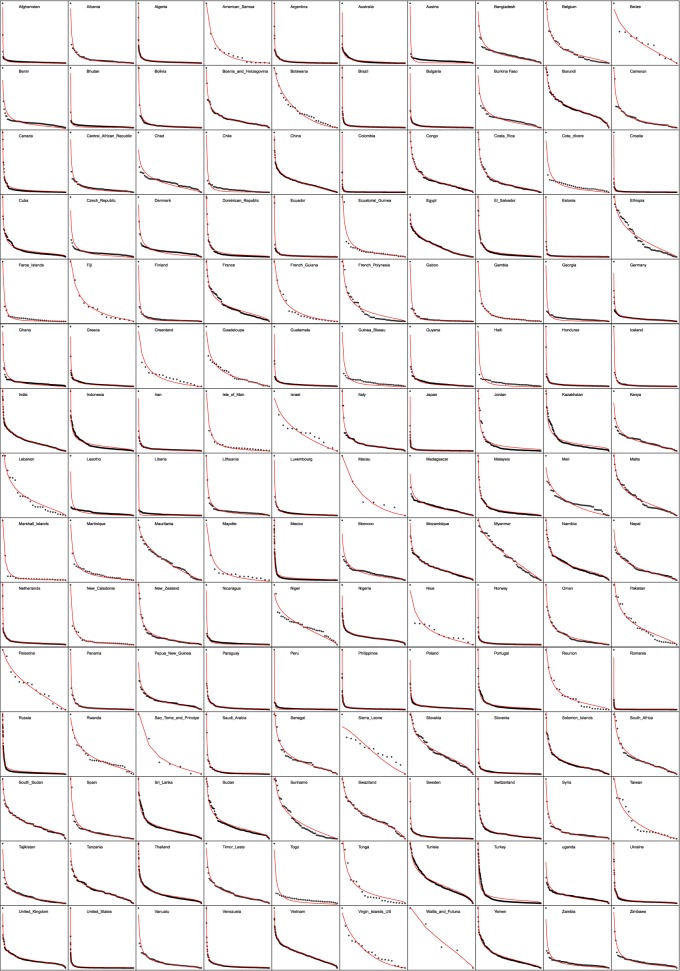
Ranked SAU population for the 150-country database. Population patterns in World’s administrative units. Dots are actual population, red lines are DGBD fits for each dataset.

In figure 10 and table 1, we present in full detail the results of the statistical analyses described in this paper. Figure 9 shows examples of outliers. Figure 10 shows ranked SAU population in logarithmic scale for our whole database, composed of 150 countries and territories. In the table, we show the results of our analysis for the set of 150 countries. For each country, the table displays the fitted *b* and *a* parameters, coefficient of determination of linear regression, number of SAUs *N*, *p*-value estimated with the bootstrap-K–S approach and logarithmic difference of Akaike’s information criterion, $\mathrm{AIC}=\log({\text{AIC}}_{\mathrm{DGBD}})-\log({\text{AIC}}_{\mathrm{power}\;\mathrm{law}})$.

## Data accessibility

The data files analysed in this work are available at https://goo.gl/xAASej.

## References

[1] López-Pellicer F, Florczyk A, Lacasta J, Zarazaga-Soria F, Muro-Medrano P 2008 Administrative units, an ontological perspective. In *Proc. of the ER 2008 Workshops (CMLSA, ECDM, FP-UML, M2AS, RIGiM, SeCoGIS, WISM) on Advances in Conceptual Modeling: Challenges and Opportunities*, ER ’08, pp. 354–363. Berlin, Heidelberg: Springer.

[2] Ma L 2005 Urban administrative restructuring, changing scale relations and local economic development in China. *Polit. Geogr.* 24, 477–497. (doi:10.1016/j.polgeo.2004.10.005)

[3] Grossman G, Lewis J 2014 Administrative unit proliferation. *Am. Polit. Sci. Rev.* 108, 196–217. (doi:10.1017/S0003055413000567)

[4] Gantner F, Waldvogel B, Meile R, Laube P 2013 The basic formal ontology as a reference framework for modeling the evolution of administrative units. *Trans. GIS* 17, 206–226. (doi:10.1111/j.1467-9671.2012.01356.x)

[5] Christenson J, Sachs C 1980 The impact of government size and number of administrative units on the quality of public services. *Adm. Sci. Q.* 25, 89–101. (doi:10.2307/2392228)

[6] Eeckhout J 2004 Gibrat’s Law for (all) cities. *Am. Econ. Rev.* 94, 1429–1451. (doi:10.1257/0002828043052303)

[7] Bettencourt L, Lobo J, Kuhnert C, West G 2007 Growth, innovation, scaling, and the pace of life in cities. *Proc. Natl. Acad. Sci. USA* 104, 7301–7306. (doi:10.1073/pnas.0610172104) 17438298 10.1073/pnas.0610172104 PMC1852329

[8] Bettencourt L, Lobo J, Strumsky D, West G 2010 Urban scaling and its deviations: revealing the structure of wealth, innovation and crime across cities. *PLoS ONE* 5, e13541 (doi:10.1371/journal.pone.0013541) 21085659 10.1371/journal.pone.0013541 PMC2978092

[9] Bettencourt L, Lobo J, Youn H 2013 The hypothesis of urban scaling: formalization, implication and challenges. (http://arxiv.org/abs/1301.5919v1)

[10] Leitao J, Miotto J, Gerlach M, Altmann E 2016 Is this scaling nonlinear? *R. Soc. Open. Sci.* 3, 150649 (doi:10.1098/rsos.150649) 27493764 10.1098/rsos.150649 PMC4968456

[11] Strano E, Sood V 2016 Rich and poor cities in Europe. An urban scaling approach to mapping the European economic transition. *PLoS ONE* 11, e0159465 (doi:10.1371/journal.pone.0159465) 27551719 10.1371/journal.pone.0159465 PMC4994959

[12] Batty M 2008 The size, scale, and shape of cities. *Science* 319, 769–771. (doi:10.1126/science.1151419) 18258906 10.1126/science.1151419

[13] Batty M 2013 A theory of city size. *Science* 3402, 1418–1419. (doi:10.1126/science.1239870) 10.1126/science.1239870 23788792

[14] Soo K 2005 Zipf’s Law for cities: a cross-country investigation. *Reg. Sci. Urban Econ.* 35, 239–263. (doi:10.1016/j.regsciurbeco.2004.04.004)

[15] Rozenfeld H, Rybski D Jr JA, Batty M, Stanley H, Makse H 2008 Laws of population growth. *PNAS* 105, 18702–18707. (doi:10.1073/pnas.0807435105) 19033186 10.1073/pnas.0807435105 PMC2596244

[16] Holmes T, Lee S 2010 Cities as six-by-six-mile squares: Zipfs Law? In *Agglomeration economics* (ed. E Glaeser), pp. 105–131. Chicago, IL: University of Chicago Press.

[17] Jiang B, Jia T 2010 Zipf’s law for all the natural cities in the United States: a geospatial perspective. *Int. J. Geograph. Info. Sci.* 25, 1269–1281. (doi:10.1080/13658816.2010.510801)

[18] Rozenfeld H, Rybski D, Gabaix X, Makse H 2011 The area and population of cities: new insights from a different perspective on cities. *Am. Econ. Rev.* 101, 2205–2225. (doi:10.3386/w15409)

[19] Courtat T, Gloaguen C, Douady S 2011 Mathematics and morphogenesis of cities: a geometrical approach. *Phys. Rev. E* 83, 036106 (doi:10.1103/PhysRevE.83.036106) 10.1103/PhysRevE.83.036106 21517557

[20] Masucci P, Stanilov K, Batty M 2013 Limited urban growth: London’s street network dynamics since the 18th century. *PLoS ONE* 8, e69469 (doi:10.1371/journal.pone.0069469) 23950895 10.1371/journal.pone.0069469 PMC3741310

[21] Levy M 2009 Gibrat’s law for (all) cities: reply. *Am. Econ. Rev.* 99, 1672–1675. (doi:10.1257/aer.99.4.1672)

[22] Eeckhout J 2009 Gibrat’s law for (all) cities: reply. *Am. Econ. Rev.* 99, 1676–1683. (doi:10.1257/aer.99.4.1676)

[23] Malevergne Y, Pisarenko V, Sornette D 2011 Testing the Pareto against the lognormal distributions with the uniformly most powerful unbiased test applied to the distribution of cities. *Phys. Rev. E* 83, 036111 (doi:10.1103/PhysRevE.83.036111) 10.1103/PhysRevE.83.036111 21517562

[24] Gabaix X 1999 Zipf’s law and the growth of cities. *Am. Econ. Rev.* 89, 129–132. (doi:10.1257/aer.89.2.129)

[25] Saichev A, Malevergne Y, Sornette D 2010 *Theory of Zipf’s law and beyond*. Berlin, Germany: Springer.

[26] Nassar I, Almsafir N, Al-Mahrouq N 2013 The validity of Gibrat’s law in developed and developing countries (2008–2013): comparison based assessment. *Proc. Soc. Behav. Sci.* 129, 266–273. (doi:10.1016/j.sbspro.2014.03.676)

[27] Vitanov N, Ausloos M 2015 Test of two hypotheses explainig the size of populations in a system of cities. *J. Appl. Stat.* 42, 2686–2693. (doi:10.1080/02664763.2015.1047744)

[28] Schaffar A, Dimou M 2012 Rank-size city dynamics in China and India 1981–2004. *Reg. Stud.* 46, 707–721. (doi:10.1080/00343404.2010.521146)

[29] Martínez-Mekler G, Martínez R, del Río MB, Mansilla R, Miramontes P, Cocho G 2009 Universality of rank-ordering distributions in the arts and sciences. *PLoS ONE* 4, e4791 (doi:10.1371/journal.pone.0004791) 19277122 10.1371/journal.pone.0004791 PMC2652070

[30] Mansilla R, Köppen E, Cocho G, Miramontes P 2007 On the behavior of journal impact factor rank-order distribution. *J. Informetrics* 1, 155–160. (doi:10.1016/j.joi.2007.01.001)

[31] del Río MB, Cocho G, Naumis G 2008 Universality in the tail of musical note rank distribution. *Physica A* 387, 5552–5560. (doi:10.1016/j.physa.2008.05.031)

[32] Li W, Miramontes P, Cocho G 2010 Fitting ranked linguistic data with two-parameter functions. *Entropy* 12, 1743–1764. (doi:10.3390/e12071743)

[33] Li W, Miramontes P 2011 Fitting ranked English and Spanish letter frequency distribution in US and Mexican presidential speeches. *J. Quant. Linguist.* 18, 337–358. (doi:10.1080/09296174.2011.608606)

[34] Li W 2012 Fitting Chinese syllable-to-character mapping spectrum by the beta rank function. *Physica A* 391, 49–53. (doi:10.1016/j.physa.2011.08.024)

[35] Li W 2012 Analyses of baby name popularity distribution in U.S. for the last 131 years. *Complexity* 18, 44–50. (doi:10.1002/cplx.21409)

[36] Li W 2013 Characterizing ranked Chinese syllable-to-character mapping spectrum: a bridge between spoken and written Chinese language. *J. Quant. Linguist.* 20, 153–167. (doi:10.1080/09296174.2013.773140)

[37] Ausloos M 2014 Two-exponent Lavalette function: a generalization for the case of adherents to a religious movement. *Phys. Rev. E* 89, 062803 (doi:10.1103/PhysRevE.89.062803) 10.1103/PhysRevE.89.062803 25019829

[38] Li X, Wang X, Zhang J, Wu L 2015 Allometric scaling, size distribution and pattern formation of natural cities. *Palgrave Commun.* 1, 15 017 (doi:10.1057/palcomms.2015.17)

[39] Cerqueti R, Ausloss M 2015 Evidence of economic regularities and disparities of Italian regions from aggregated tax income size data. *Physica A* 421, 187–207. (doi:10.1016/j.physa.2014.11.027)

[40] Cerqueti R, Ausloss M 2015 Cross rankings of cities and regions: population versus income. *J. Stat. Mech.* 2015, P07002 (doi:10.1088/1742-5468/2015/07/P07002)

[41] Ausloss M, Cerqueti R 2016 A universal rank-size law. *PLoS ONE* 11, e0166011 (doi:10.1371/journal.pone.0166011) 27812192 10.1371/journal.pone.0166011 PMC5094590

[42] Naumis G, Cocho G 2007 The tails of rank-size distributions due to multiplicative processes: from power laws to stretched exponentials and beta-like functions. *New J. Phys.* 9, 286 (doi:10.1088/1367-2630/9/8/286)

[43] del Río MB, Cocho G, Mansilla R 2011 General model of subtraction of stochastic variables. Attractor and stability analysis. *Physica A* 390, 154–160. (doi:10.1016/j.physa.2010.09.035)

[44] Alvarez-Martinez R, Martinez-Mekler G, Cocho G 2011 Order-disorder transition in conflicting dynamics leading to rank-frequency generalized beta distributions. *Physica A* 390, 120–130. (doi:10.1016/j.physa.2010.07.037)

[45] Li W, Fontanelli O, Miramontes P 2016 Size distribution of function-based human gene sets and the split-merge model. *R. Soc. Open. Sci.* 3, 160275 (doi:10.1098/rsos.160275) 27853602 10.1098/rsos.160275 PMC5108952

[46] Sornette D 2006 *Critical phenomena in natural sciences*, 2nd edn Berlin, Germany: Springer.

[47] Sidak Z, Sen P, Hajek J 1999 *Theory of rank tests*. 2nd edn. San Diego, CA: Academic Press.

[48] Fontanelli O, Miramomtes P, Yang Y, Cocho G, Li W 2016 Beyond Zipf’s law: the Lavalette rank function and its properties. *PLoS ONE* 11, e0163241 (doi:10.1371/journal.pone.0163241) 27658296 10.1371/journal.pone.0163241 PMC5033250

[49] Lahehrre J, Sornette D 1998 Stretched exponential distributions in nature and economy: fat tails with characteristic scales. *Eur. Phys. J. B* 2, 525–539. (doi:10.1007/s100510050276)

[50] Pericchi L, Torres D 2011 Quick anomaly detection by the Newcom-Benford law, with applications to electoral processes data from the USA, Puerto Tico and Venezuela. *Stat. Sci.* 26, 502–516. (doi:10.1214/09-STS296)

[51] Clauset A, Shalizi C, Newman M 2009 Power-law distributions in empirical data. *SIAM Rev.* 51, 661–703. (doi:10.1137/070710111)

[52] Bates D, Watts D 2008 *Nonlinear regression analysis and its applications*, 2nd edn New York, NY: John Wiley and Sons.

[53] Gill PE, Murray W, Wright M 1997 *Practical optimization*. San Diego, CA: Academic Press.

[54] Colquhoun D 2014 An investigation of the false discovery rate and the misinterpretation of *p*-values. *R. Soc. Open. Sci.* 1, 140216 (doi:10.1098/rsos.140216) 26064558 10.1098/rsos.140216 PMC4448847

[55] Spiess A, Neumeyer N 2010 An evaluation of *R*^2^ as an inadequate measure for nonlinear models in pharmacological and biochemical research: a Monte Carlo approach. *BMC Pharmacol.* 10, 6 (doi:10.1186/1471-2210-10-6) 20529254 10.1186/1471-2210-10-6 PMC2892436

[56] Fontanelli O 2017 The discrete generalized beta distribution: three study cases. Ph.D. thesis, UNAM. See http://132.248.9.195/ptd2017/enero/301630072/Index.html.

[57] Cristelli M, Batty M, Pietronero L 2012 There is more than a power Law in Zipf. *Sci. Rep.* 2, srep00812 (doi:10.1038/srep00812) 10.1038/srep00812 PMC3492871 23139862

[58] Madry C, Kaczmarek-Khubnaia J 2016 Historical determinants of regional divisions of Georgia and their implications for territorial governance. *Quaestiones Geographicae* 2, 131–139. (doi:10.1515/quageo-2016-0021)

[59] Turner B 1999 Reino de Espana (Kingdom of Spain). In *The Statesman’s Yearbook 2000*, pp. 1445–1459, London, UK: Palgrave Macmillan.

[60] Ausloss M 2015 New region planning in France? Better order or more disorder? *Entropy* 17, 5695–5710. (doi:10.3390/e17085695)

[61] Peng G 2010 Zipf’s law for Chinese cities: rolling sample regressions. *Physica A* 389, 3804–3813. (doi:10.1016/j.physa.2010.05.004)

[62] Giesen K, Zimmermann A, Suedekum J 2010 The size distribution across all cities. Double Pareto lognormal strikes. *J. Urban Econ.* 68, 129–137. (doi:10.1016/j.jue.2010.03.007)

